# Microencapsulated and Conventional Clomazone Formulations: Dynamics in Soil, Crop Residue, and Soybean

**DOI:** 10.1021/acsomega.6c01359

**Published:** 2026-06-11

**Authors:** Jéssica Alves Bonamichi, Roberto Estêvão Bragion de Toledo, Grazielle Rodrigues Araujo, Ana Karollyna Alves de Matos, Ivana Paula Ferraz Santos de Brito, Diego Gonçalves Alonso, Richard Feliciano, Ricardo Alcántara-de la Cruz, Edivaldo D. Velini, Caio A. Carbonari

**Affiliations:** † Center for Advanced Research in Weed Science, Department of Plant Protection, School of Agriculture, São Paulo State University (UNESP), Botucatu 18610-034, Brazil; ‡ Ourofino Agrociência, Uberaba 38044-750, Brazil; § Department of Agronomy, 28120 Federal University of Viçosa, Viçosa 36570-900, Brazil; ∥ Bioativa - Strategic Research in Biosciences, Botucatu 18605-525, Brazil

## Abstract

Clomazone is widely used in no-tillage systems, where volatilization and leaching can reduce its effectiveness. Microencapsulation has been proposed to improve herbicide stability and persistence. This study evaluated the behavior of two microencapsulated (ME) and two emulsifiable concentrate (EC) commercial clomazone formulations under controlled conditions in soil, corn residue, and soybean plants. In soil, the evaluated ME formulations showed low initial leaching but a gradual increase over time, reaching 42.3% (ME-1) and 36.9% (ME-2) at 30 days after application (DAA). In contrast, the EC formulations exhibited high initial leaching at 1 DAA (22% for EC-1 and 20% for EC-2), followed by lower cumulative losses than those observed for the ME formulations. In corn residue, clomazone recovery was higher for the ME formulations (40.8 μg for ME-1 and 27.6 μg for ME-2) compared with the EC formulations (11.5 μg for EC-2 and 5.8 μg for EC-1). In soybean plants, the ME formulations retained more than 90% of clomazone on the leaf surface, with 78–86% remaining encapsulated, whereas the EC formulations showed higher internal absorption (up to 69%). No significant phytotoxicity was observed in soybean for any formulation. Overall, the evaluated ME formulations modified the environmental fate of clomazone and plant exposure pathways compared to the tested EC formulations. These results indicate differences in environmental behavior and crop selectivity in no-tillage systems but are limited to the clomazone formulations evaluated.

## Graphical abstract

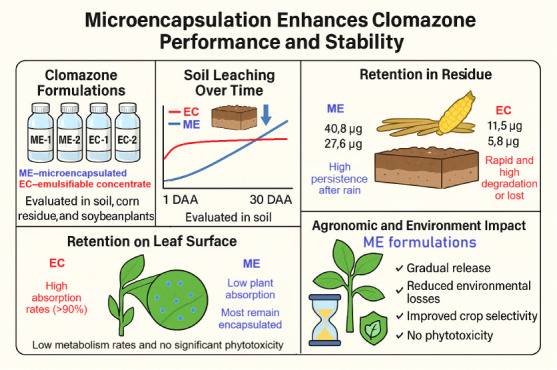

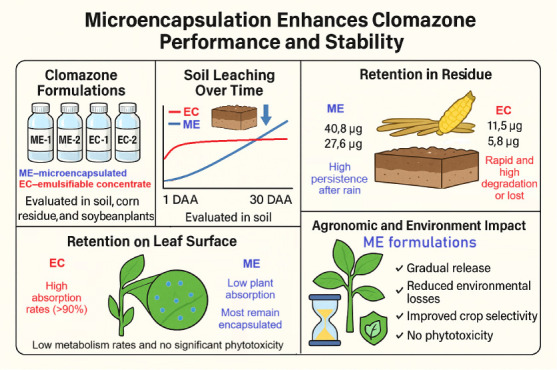

## 1 Introduction

Brazil leads the world in soybean production and export, with a cultivated area of 47.4 million hectares and an average yield of 3,903 kg ha^–1^ in the 2024/25 harvest.^1^ Part of the success of modern soybean cultivation is due to the adoption of efficient technologies, such as the use of herbicide-tolerant transgenic cultivars adapted to producing regions and the no-tillage system. This production system was adopted on approximately 33 million hectares in Brazil in 2018, representing an increase of 84.9% from 2006 to 2017.^2–4^ A widely used example is the soybean-corn succession system, where soybean is grown in the summer and second-crop corn in the fall/winter, maintaining soil cover and optimizing resource use throughout the year.^5^

The no-tillage system, characterized by sowing over crop residues or desiccated plants, is considered more environmentally and economically sustainable than conventional tillage.^3,4^ However, weed management in this system requires special attention, as the presence of crop residue increases the complexity of weed control.^6^ Although crop residue helps reduce weed infestation, it interferes with the dynamics of preemergence herbicides applied to the soil.^7,8^ This occurs because herbicide behavior in residue-covered soils differs from that in conventionally tilled soils.^3^ The residue acts as a physical barrier, hindering herbicide movement to the soil and potentially favoring losses through volatilization, photolysis, and thermal degradation. These processes may occur simultaneously or individually until the herbicide is transported to the soil by rainwater or irrigation.^9^

Clomazone (2-(2-chlorobenzyl)-4,4-dimethyl-1,2-oxazolidin-3-one), belonging to the isoxazolidinone group, is used to control important annual grasses and some broadleaf weeds.^10^ This herbicide acts by inhibiting the enzyme 1-deoxy-d-xylulose-5-phosphate synthase (DXP synthase), essential for carotenoid synthesis. DXP inhibition can lead to lipid peroxidation and damage to chlorophyll, proteins, and plant membranes.^11,12^ Clomazone has a water solubility of 1,100 mg L^–1^ at 25 °C and moderate volatility, with a vapor pressure of 1.92 × 10^–2^ Pa at the same temperature.^13^ These characteristics can lead to significant herbicide losses after application.^14^ In soil, clomazone can leach and affect sensitive crops.^10,11^ Under high-temperature conditions common on residue surfaces, these losses intensify, compromising weed control and increasing environmental contamination.^15^

Microencapsulated (ME) formulations have emerged as a strategy to mitigate herbicide losses resulting from volatilization, leaching, photolysis, and thermal degradation—challenges that are particularly prevalent in older conventional formulations, such as emulsifiable concentrate (EC) formulations of clomazone.^15^ The primary objective of ME formulations is to reduce off-site migration and unintended effects on nontarget organisms by enabling the controlled release of active ingredients.^16^ This controlled release minimizes spray drift and phytotoxicity in sensitive crops like soybean, as well as decreases herbicide losses through volatilization and leaching.^17–19^

Several studies have demonstrated that both in solo applications and in combination with other active ingredients, ME formulations of clomazone preserve its herbicidal properties, ensuring effective, consistent, and reliable control of a wide range of weed species, while maintaining a high level of crop selectivity.^14,20,21^ Despite technological advances, few studies have evaluated the behavior of different ME and EC clomazone formulations in soybean crops, residues of the preceding crop—usually from corn—and soil, that is, in a broader agricultural context. Therefore, this study aimed to evaluate the dynamics of ME formulations of clomazone in soil, corn residue, and soybean plants in postemergence applications, compared to older EC formulations of this herbicide.

## 2 Materials and Methods

### 2.1 Experimental Setup

Three experiments were conducted to evaluate the behavior of different clomazone formulations in (i) medium-textured soil under simulated rainfall at different times after application; (ii) corn residue with varying rainfall amounts; and (iii) soybean plants in postemergence applications with different intervals between application and rainfall.

#### 2.1.1 Dynamics of Clomazone Formulations in Soil

Four clomazone formulations were tested at a dose of 720 g a.i. ha^–1^: two microencapsulated [Kaivana (ME-1) (Ourofino Química S.A.) and Reator (ME-2) (FMC Química do Brasil Ltda.)] and two emulsifiable concentrates [Gamit Star (EC-1) (Ourofino Química S.A.) and GrandeBR (EC-2) (FMC Química do Brasil Ltda.)]. These commercial formulations differ in their formulation technologies; however, detailed compositional information (e.g., solvent systems and encapsulation matrices) is proprietary to the manufacturers and therefore not publicly available. Thus, the present study focuses on the comparative behavior of the formulations under experimental conditions rather than their specific chemical composition; therefore, interpretations should be limited to the comparative behavior of the evaluated formulations and not extrapolated to broader formulation categories.

The design was completely randomized with four replicates. Experimental units consisted of 80 mL containers filled with 32 g of soil (Table 1). A 32 μL aliquot of the herbicide solution (equivalent to 720 g a.i. ha^–1^ in 200 L ha^–1^) was added, and samples were homogenized and transferred to polypropylene capsules containing a filter and 5 g of washed sand. Samples were kept outdoors under sunlight, protected in a quartz structure with forced air circulation. Simulated rainfall was applied in two stages—11.3 mm (saturation) and 33.9 mm (leaching)—at 1, 3, 7, 14, and 30 days after application (DAA). The system consisted of a metal frame with eight DG9505EVS nozzles (TeeJet, Wheaton, IL, USA), spaced 10 cm apart and positioned 1.4 m above the samples. Leachate from each period was collected and stored for subsequent chromatographic analysis.

**Table 1:** Physicochemical Analysis of the Soil Used in the Experiment^a^

Ph (CaCl_2_)	M.O. (g dm^–3^)	P (mg dm^–3^)	H + Al^3+^ (mmol_c_ dm^–3^)	K (mmol_c_ dm^–3^)	Ca (mmol_c_ dm^–3^)	Mg (mmol_c_ dm^–3^)	SB (mmol_c_ dm^–3^)	CEC (mmol_c_ dm^–3^)	V (%)
4,1	19	9	52	1, 2	6	6	13	65	20

Sand	Clay	Silt	Soil texture
587 g kg^–1^	317 g kg^–1^	96 g kg^–1^	Medium

a M.O. = organic matter; SB = sum of bases; CEC = cation exchange capacity; V = base saturation (%).

#### 2.1.2 Dynamics in Corn Residue

The same four clomazone formulations, applied at the same dose as in the previous experiment, were applied to corn residue (6 t ha^–1^), cut into 0.5 × 0.5 cm fragments and placed in polypropylene laminated capsules with a diameter of 4.5 cm. The experiment was completely randomized with four replicates. Application was carried out with a stationary sprayer equipped with four flat fan nozzles XR110.02VS (TeeJet, Wheaton, IL, USA), spaced 0.5 m apart and positioned 0.5 m above the target surface. The equipment operated at 150 kPa pressure and a travel speed of 3.6 km h^–1^, delivering a spray volume of 200 L ha^–1^. After 24 h, experimental units received simulated rainfall of 10, 20, 35, 50, and 100 mm. The solutions that passed through were collected for subsequent analysis.

#### 2.1.3 Dynamics in Soybean Plants

Soybean seeds of cultivar NA5909RG were sown in 1.7 L pots containing 0.5 kg of commercial substrate, composed of single superphosphate, potassium nitrate, peat, expanded vermiculite, and pine bark (pH 5.8 ± 0.5). After sowing, thinning was performed to maintain only one plant per pot. Treatments with the four clomazone formulations (720 g a.i. ha^–1^) were applied at the V4 growth stage in a completely randomized design with eight replicates. Treated plants were subjected to simulated rainfall (20 mm) at 1, 3, and 7 DAA and were kept in plastic trays to collect runoff solution for later herbicide content analysis. Some experimental units were washed at the same intervals to quantify external clomazone and its metabolites (hydroxyclomazone and ketoclomazone). The remaining units were maintained for dry matter determination of leaves in a forced-air circulation oven at 55 °C for 20 DAA.

### 2.2 Extraction and Chromatographic Analysis of Clomazone in Different Matrices

#### 2.2.1 Soil and Residue Samples

For EC formulations, 2 mL of leachate solutions were filtered through PVDF membranes (0.45 μm; 13 mm diameter). Then, 0.6 mL of the filtrate was transferred to vials containing an equal volume of methanol, composing the chromatographic phase [methanol:water (50:50, v/v)]. For ME formulations, 2 mL of solution was filtered through a 0.45 μm porous membrane to separate the free active ingredient and retain the herbicide capsules. This filtration step allowed the separation of dissolved clomazone from the microencapsulated fraction, as the microcapsules are substantially larger than the membrane pore size. The filtered solution was used to quantify free clomazone. Filters containing the retained capsules underwent backwashing with 2 mL methanol, followed by sonication for 30 min to break the capsules and release the encapsulated active ingredient. The resulting solution was diluted in 0.6 mL ultrapure water and stored in vials for chromatographic analysis (mobile phase: methanol:water, 50:50, v/v).

The amount of clomazone recovered in experimental units was considered the amount of herbicide that effectively reached the residue. Leached concentrations were expressed in μg per experimental unit.

#### 2.2.2 Plant Samples—Analysis of External and Internal Clomazone Contents

Each leaf sample was washed twice with 150 mL of deionized water to quantify external clomazone. The resulting washing solution was homogenized, and 10 mL was stored for analysis. External herbicide content was considered as the sum of values quantified in washing water and simulated rainfall. Washed plant material was frozen at −80 °C for subsequent internal clomazone quantification.

Internal quantification of the herbicide and its metabolites was performed by macerating frozen leaf material using liquid nitrogen. Aliquots of 0.2 g of macerate were transferred to 15 mL Falcon tubes containing 10 mL of extraction solution [methanol:water (80:20, v/v)]. Samples were sonicated for 30 min, followed by centrifugation (Hettich Zentrifugen centrifuge) at 5,000 rpm for 5 min. The supernatant was filtered through a PVDF membrane (0.45 μm; 13 mm) and transferred to vials for chromatographic analysis. Internal content analysis considered the sum of herbicide and metabolite concentrations.

#### 2.2.3 Analytical System and Chromatographic Conditions

Quantification of clomazone and its metabolites in soil, residue, and soybean plant samples was carried out using liquid chromatography coupled with tandem mass spectrometry (LC-MS/MS). The analyses were performed on a high-performance liquid chromatograph (LC-40D XR) coupled to a triple quadrupole mass spectrometer (LCMS-8060NX), both from Shimadzu. The total run time was 11 min, with retention times of 3.80 min for clomazone, 3.41 min for hydroxyclomazone, and 3.50 min for ketoclomazone. Chromatographic separation was achieved using a Synergi Hydro-RP analytical column (2.5 μm, 100 Å, 50 × 4.6 mm) at 40 °C, with a 20 μL injection volume and a mobile phase flow rate of 0.6 mL min^–1^. The mobile phase consisted of water with 0.1% acetic acid (phase A) and methanol with 0.1% acetic acid (phase B), applied under the following gradient program: 0–1 min, 40% B; 1–6 min, 95% B; 6–8 min, 40% B; followed by equilibration until 11 min. Analytical calibration curves, determination coefficients (r^2^) for each compound, molecular masses, characteristic ion fragments, and representative chromatograms are provided in Tables S1, S2, and Figure S1, respectively.

### 2.3 Statistical Data Analysis

Leaching data from rainfall volumes in soil and residue were modeled using the Mitscherlich nonlinear regression equation:,^22^

$$Y=a\left[1-10^{-c\left(X+b\right)}\right]$$

where Y represents the cumulative amount of herbicide leached and X denotes the number of days after application. In this model, parameter a represents the estimated maximum leaching value, b reflects the displacement of the curve along the X-axis (indicating when leaching begins to intensify), and c is the slope coefficient that defines the rate of leaching. The nonlinear regression model was used to describe cumulative leaching dynamics, and statistical procedures were selected to adequately represent the experimental patterns while maintaining a parsimonious analytical framework.

Clomazone data were analyzed using factorial analysis of variance (ANOVA), considering formulation and time as fixed factors. When significant effects were detected, means were compared using Tukey’s test (*p* ≤ 0.05). For external, internal, and total clomazone contents (expressed in μg g^–1^ dry mass), as well as plant dry mass, data were summarized using appropriate descriptive statistics.

Statistical analyses were performed using Statistix software, and data visualization was conducted with SigmaPlot 12.5.

## 3 Results

### 3.1 Dynamics of Clomazone Formulations in Soil

Clomazone leaching differed between the two formulation types (Figure 1), with significant effects of formulation, time, and their interaction (*p* ≤ 0.05) (Table S3). The ME formulations showed low leaching at 1 DAA (5.3% for ME-1 and 4.5% for ME-2), but values increased gradually over time, peaking at 7 DAA (14.5%) (Figure 1A). Total leaching reached 42.3% for ME-1 and 36.9% for ME-2 by 30 DAA (Figure 1B), indicating a gradual release of the active ingredient. In contrast, the EC formulations showed a pronounced initial leaching, with 22% (EC-1) and 20% (EC-2) at 1 DAA (Figure 1A). After this initial period, leaching remained below 5% at each interval, resulting in total accumulated leaching of 34% (EC-1) and 29.8% (EC-2) by 30 DAA (Figure 1B).

**Figure 1:**
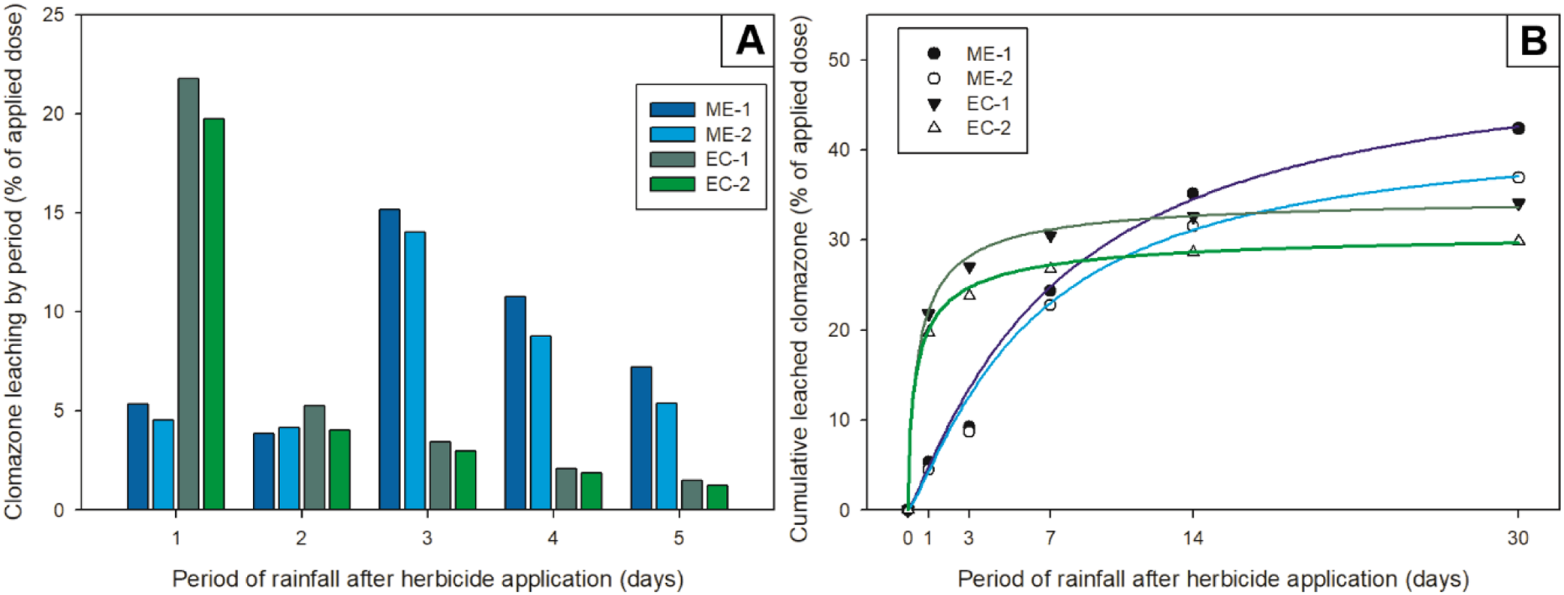
Leaching of microencapsulated (ME) and conventional emulsifiable concentrate (EC) formulations of clomazone in medium-textured soil, as a function of the rainfall occurrence period after application. A) Clomazone leached in each evaluated period; B) Cumulative clomazone leaching. Statistical effects of formulation, period, and their interaction were evaluated by two-way ANOVA (*p* ≤ 0.05), with detailed results presented in Table S3 and regression parameters shown in Table 1.

The Mitscherlich model adequately described the cumulative leaching dynamics for all formulations, with high coefficients of determination (r^2^ > 0.99) and significant F values (*p* ≤ 0.01), indicating a good fit of the model to the observed (Table 2). Differences in parameter estimates were observed between the evaluated formulations.

**Table 2:** Parameter Estimates of the Mitscherlich Models Fitted to the Correlation between the Total Accumulated Amount of Clomazone Leached from Medium-Textured Soil (% of the Applied Dose) as a Function of Rainfall Occurrence Periods after the Application of the Formulations^a^

	Parameter estimates				
Formulation	a	b	c	r^2^	F value
ME-1	45.2118	0	0.00456	0.9972	706.41**
ME-2	39.0327	0	0.00503	0.9970	664.85**
EC-1	31.6169	0	0.4572	0.9954	431.81**
EC-2	27.6462	0	0.4953	0.9951	409.31**

a Significant by the F-test at *p* ≤ 0.01.

### 3.2 Dynamics in Corn Residue

After the application of 100 mm of simulated rainfall, the ME formulations showed greater recovery of clomazone: 40.8 μg per experimental unit for ME-1 and 27.6 μg for ME-2 (Figure 2A, B). In comparison, the EC formulations recovered a maximum of 11.5 μg (EC-2) and 5.8 μg (EC-1) per experimental unit (Figure 2C, D). Regression analysis using the Mitscherlich model showed high goodness-of-fit for clomazone dynamics in corn residue (r^2^ > 0.99; Table 3), indicating consistent model performance across the evaluated conditions. Higher recovery values were observed for the evaluated ME formulations across all rainfall amounts. For example, for ME-1, about 85% of the clomazone remained encapsulated at 1 DAA, whereas only 15% was present in the soil solution (Figure 2A). In contrast, the EC formulations had lower recovery values of clomazone throughout the evaluated conditions (Figure 2C, D).

**Figure 2:**
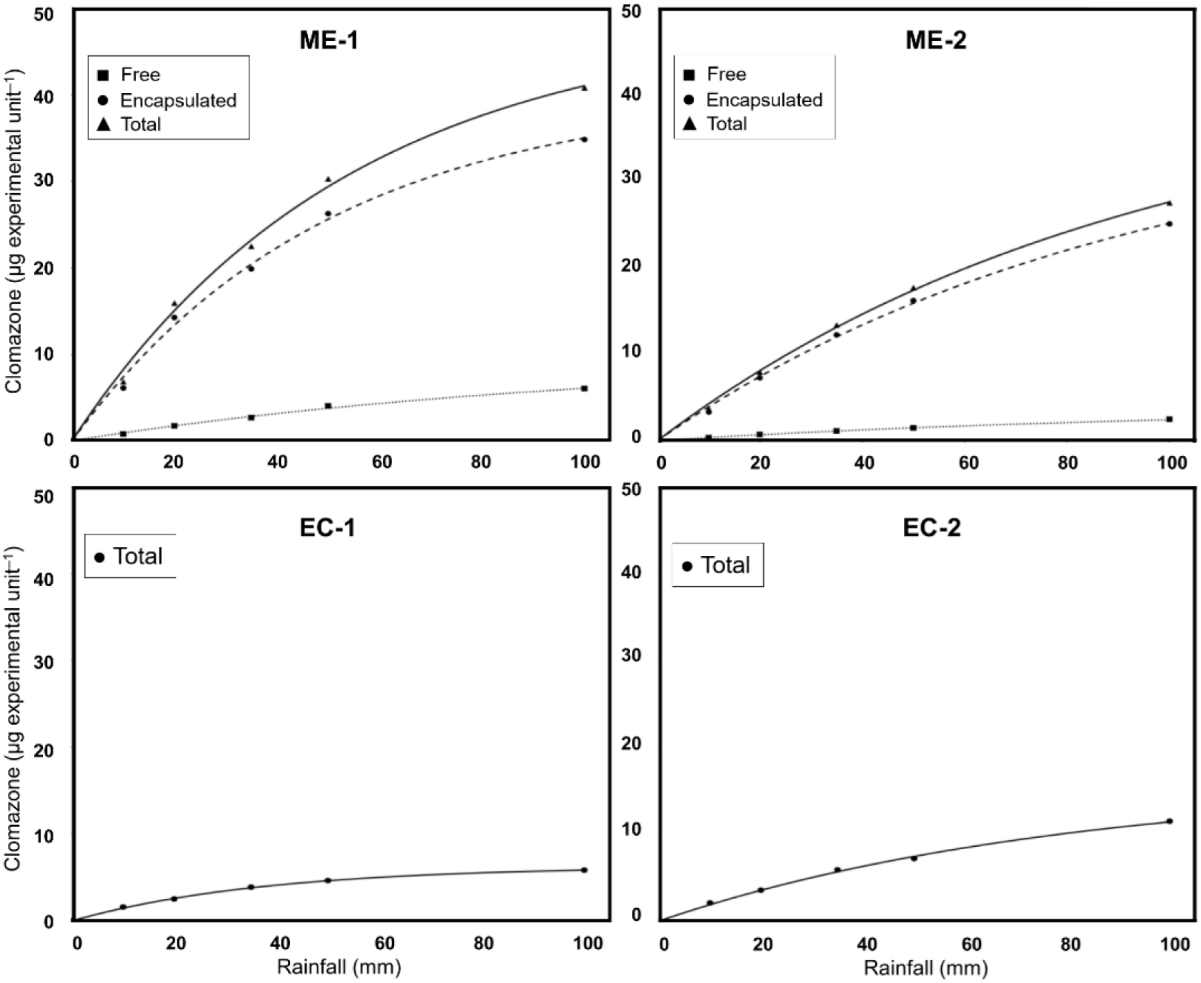
Amounts of clomazone leached from corn straw (free, encapsulated, or total) at 24 h after application, under simulated rainfall at different intensities (mm), for microencapsulated (ME) and conventional emulsifiable concentrate (EC) formulations.

**Table 3:** Parameter Estimates of the Mitscherlich Models Fitted to the Correlation between the Amount of Clomazone Leached from Straw as a Function of Simulated Rainfall Depths (mm)^a^ ^,^ ^b^

		Parameter estimates				
Formulation	Form	a	b	c	r^2^	F value
	Free	9.7546	0	0.00421	0.9979	938.97**
ME-1	Encapsulated	40.4215	0	0.00873	0.9987	1616.02**
	Total	48.6974	0	0.00804	0.9988	1655.64**
	Free	3.9249	0	0.00412	0.9996	4980.91**
ME-1	Encapsulated	37.9511	0	0.00472	0.9995	4051.1**
	Total	41.9977	0	0.00465	0.9996	4830.47**
EC-1	Total	6.2777	0	0.0116	0.9998	9182.66**
EC-2	Total	15.9273	0	0.00547	0.9996	3781.97**

a 24 h after the application of different formulations.

b Significant by the F-test at *p* ≤ 0.01.

### 3.3 Dynamics in Soybean Plants

The ME formulations showed higher total contents of clomazone compared to the EC formulations at all evaluation periods, with significant effects of formulation type for external and internal clomazone contents (*p* ≤ 0.05). External herbicide levels decreased over time in all formulations, especially in EC-1 and EC-2, which already showed low levels at 1 DAA (10 μg g^–1^ and 15 μg g^–1^, respectively). In contrast, the ME formulations maintained high external clomazone levels over time, with 330 μg g^–1^ for ME-1 and 469 μg g^–1^ for ME-2 at 1 DAA. On average, approximately 93% (ME-1) and 97% (ME-2) of clomazone remained in the external form throughout the evaluated period (Figure 3).

**Figure 3:**
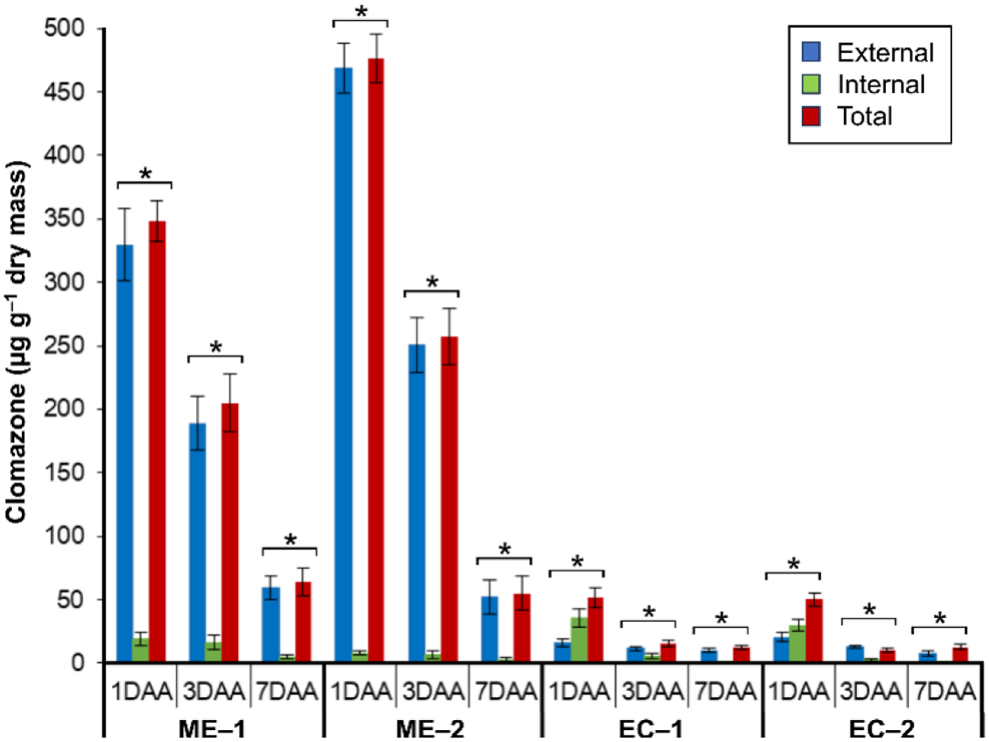
External, internal, and total levels of clomazone (μg g^–1^ dry mass) in soybean plants at 1, 3, and 7 days after application (DAA) of microencapsulated (ME) and conventional emulsifiable concentrate (EC) formulations. Error bars represent standard deviation. Brackets indicate comparisons within each evaluation time, and * denotes differences according to two-way ANOVA followed by Tukey’s test (*p* ≤ 0.05).

Clomazone distribution between internal and external fractions differed significantly between formulation types (*p* ≤ 0.05). The ME formulations exhibited low internal concentrations of clomazone in soybean plants. On the other hand, the EC formulations had, on the first day of evaluation, 69% and 59%, respectively, of their total contents inside the plants (Figure 4A). Regarding external herbicide, about 78% and 86% of the clomazone in formulations ME-1 and ME-2, respectively, remained encapsulated on the leaf surface at 1 DAA. Over time, these values decreased, with corresponding changes in the distribution between external and internal fractions (Figure 4B).

**Figure 4:**
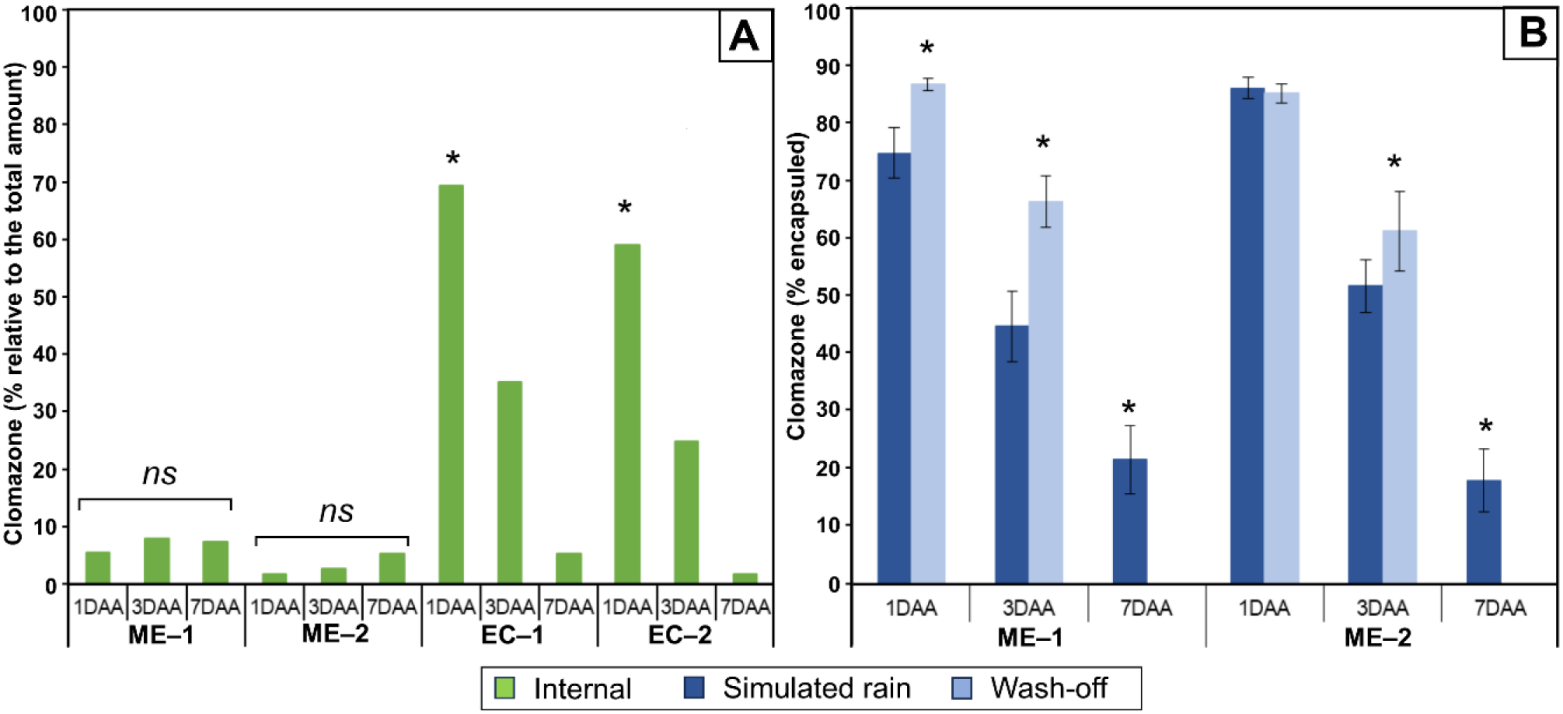
A) Internal levels of clomazone (% of the total applied) in soybean plants at 1, 3, and 7 days after application (DAA) of the microencapsulated (ME) and emulsifiable concentrate (EC) formulations. B) Percentage of encapsulated clomazone quantified externally in soybean plants at 1, 3, and 7 DAA of the ME formulation, based on the amount recovered from simulated rainfall and plant material washings. In panel A, brackets indicate comparisons between formulation types within each evaluation time, with “ns” denoting nonsignificant differences. In panel B, comparisons are made between simulated rainfall and wash-off at each evaluation time, and * denotes differences according to two-way ANOVA followed by Tukey’s test (*p* ≤ 0.05).

No differences in clomazone metabolite concentrations were observed between formulations (*p* ≥ 0.05). At 1 DAA, the EC formulations showed residual concentrations of hydroxyclomazone, while ketoclomazone was not detected in any sample (Figure 5). Similarly, dry mass was not affected by formulation type (*p* ≥ 0.05), despite numerical reductions of up to 14% in EC treatments, but these reductions did not compromise plant development (Figure 6).

**Figure 5:**
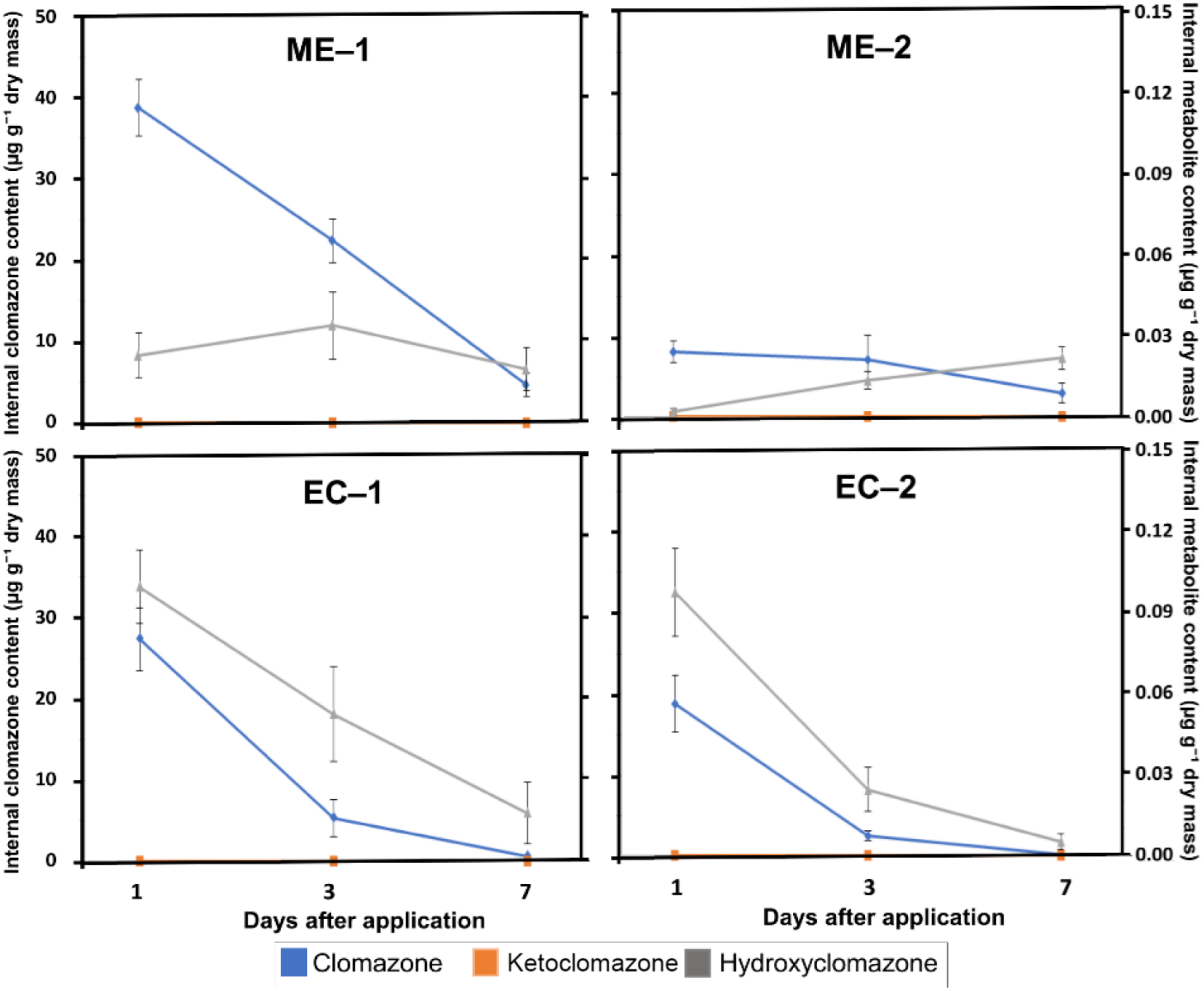
Internal levels of microencapsulated (ME) and conventional emulsifiable concentrate (EC) formulations of clomazone and its metabolites hydroxyclomazone and ketoclomazone at 1, 3, and 7 days after application (DAA) in soybean plants.

**Figure 6:**
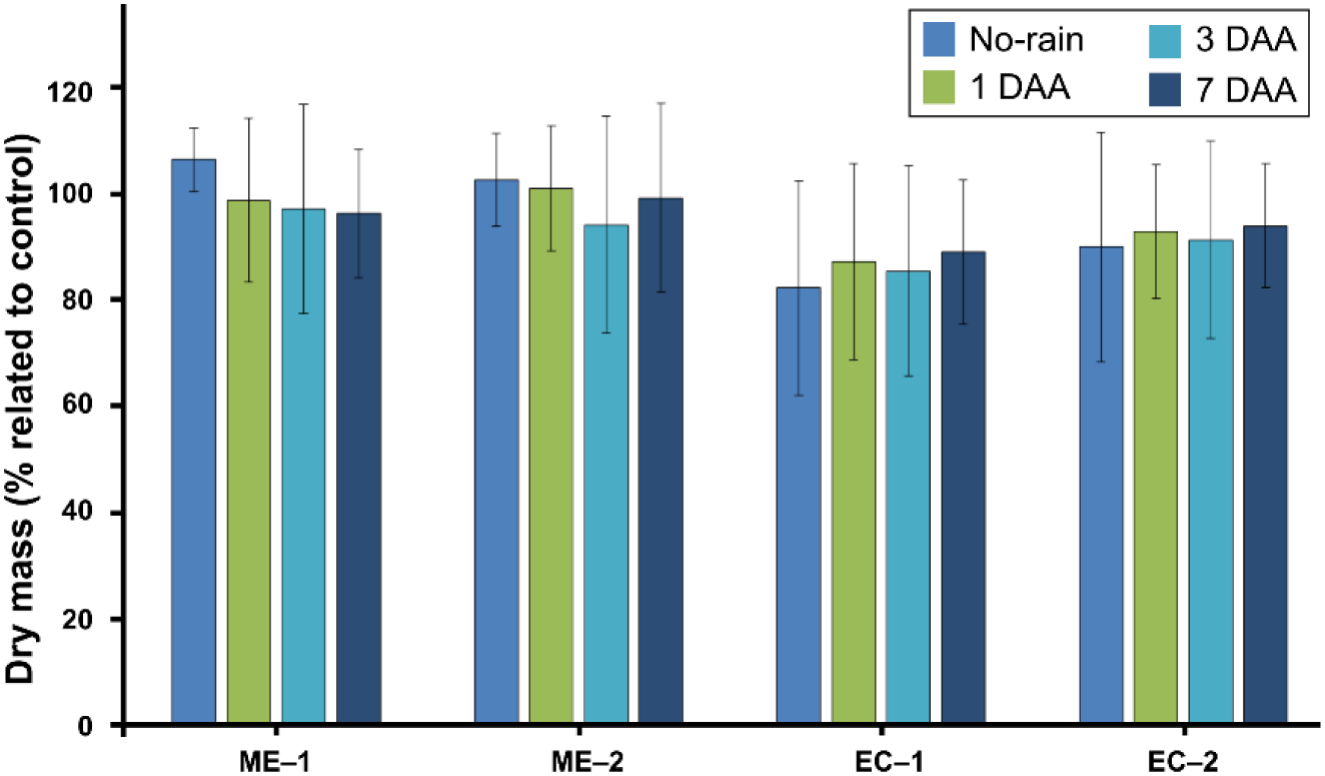
Dry mass of soybean plants (% relative to the untreated control), collected 20 days after application (DAA), using microencapsulated (ME) and conventional emulsifiable concentrate (EC) formulations of clomazone subjected to simulated rainfall at 1, 3, and 7 DAA, as well as a treatment without simulated rain. No significant differences were observed between treatments according to two-way ANOVA (*p* ≤ 0.05).

## 4 Discussion

The ME clomazone formulations exhibited a distinct behavior to EC formulations, in soil and crop residue dynamics, as well as in foliar absorption in soybean plants. These differences were consistent across the evaluated experimental conditions. Although similar trends were observed among formulations grouped as ME or EC, caution is required when attributing these differences exclusively to formulation technology, as compositional details are not available and product-specific characteristics may also contribute. ME formulations showed greater herbicide retention in the soil, even after intense simulated rainfall (100 mm), delaying the clomazone availability in the soil solution. This behavior aligns with the principles of controlled-release pesticide formulations, in which the polymeric matrix acts as a diffusion barrier regulating the release of the active ingredient in response to environmental drivers such as moisture and temperature.^23^

On the first day of evaluation, higher mobility of clomazone was observed in the EC formulations compared to ME formulations. In EC formulations, clomazone is released into the soil solution immediately after application, increasing its exposure to dissipation processes such as volatilization and surface losses, thereby reducing the amount of herbicide available for subsequent downward movement.^15^ In contrast, the progressive rupture of microcapsules results in a delayed and sustained release, which may explain the cumulative increase in leaching observed for ME formulations at later evaluation times.

The release of microcapsule contents is favored by factors such as temperature, pH, solubility, biodegradation, and mechanical disturbances.^24^ Under field-like conditions, diurnal thermal variation and rainfall events progressively weaken the polymeric matrix, allowing gradual diffusion of the active ingredient. Thus, encapsulation promotes a more gradual release profile, which may contribute to differences in residual availability compared to EC formulations.^19,25^ This temporal redistribution of herbicide availability may influence agronomic performance by better synchronizing clomazone presence in the soil with the critical weed emergence period. During early weed establishment, clomazone inhibits carotenoid biosynthesis, compromising chlorophyll protection against photooxidative damage.^26^ As a result, emerging seedlings exhibit bleaching symptoms, impaired photosynthesis, and reduced growth.

The recovery rate of clomazone on corn residue was higher in the ME formulations, particularly as precipitation volume increased. The high proportion of encapsulated clomazone retained on the residue surface is consistent with reduced wash-off under increasing rainfall.^14^ In a study with sugarcane residue, ME formulations maintained over 80% recovery of the herbicide even after 100 mm rainfall, with a large portion of clomazone still encapsulated.^15^ These findings are consistent with previous reports

Comparison between the evaluated formulations showed that the availability of free clomazone is higher in EC formulations. However, this availability may fall below effective levels due to the high losses, which may be associated with clomazone’s physicochemical properties, including vapor pressure.^12,21^ Herbicides with high vapor pressure are particularly prone to volatilization losses in the first hours or days after application, especially when retained on crop residues exposed to elevated temperatures.^27^ As a result, only a fraction of the dose effectively reaches the soil, potentially reducing weed control.^28^

The presence of crop residue on the soil surface reduces solar radiation incidence and soil temperature,^15,29,30^ but the residue itself may reach high temperatures under direct radiation, intensifying volatilization processes of freely available herbicides retained on the surface. In exposed areas, photodegradation and volatilization of clomazone may be intensified, since volatilization increases proportionally with temperature.^31^ Under these conditions, higher herbicide losses may occur, influencing the amount of product reaching the soil and its subsequent availability for weed control.

The internal concentration of clomazone in soybean leaves was higher in EC formulations. This pattern suggests that, being in emulsion and free form, EC formulations may be absorbed more rapidly by leaves. Additionally, the oil-based nature of these formulations enhances cuticular penetration, which may reduce the herbicide selectivity and crop safety.^32^ This is consistent with the higher proportion of internal clomazone observed shortly after application in EC-treated soybean plants. Over time, internal concentrations decreased, likely due to translocation and metabolic processes.^33,34^ After foliar absorption, clomazone can be translocated via xylem, following the transpiration stream toward growing tissues, while metabolic transformation and volatilization contribute to its dissipation.^32,33^ The ME formulations showed higher recovery of the active ingredient compared to EC after simulated rain. This behavior is particularly relevant for postemergence applications, as it reduces the risk of acute phytotoxicity while still allowing gradual herbicide transfer to the soil via rainfall.^17,21,35^

Clomazone metabolism in soybean did not differ between formulations, suggesting that the herbicide remains mostly in its original form. In addition, clomazone has relatively low root absorption and limited metabolism.^10^ In ME formulations, reduced internal contents are consistent with lower internal exposure and more gradual availability of the active ingredient.^32,36^ Since only the metabolite ketoclomazone effectively inhibits DXP synthase, while clomazone itself has limited direct phytotoxicity, differences in internal availability may influence metabolite formation and crop selectivity.^26^

Soybean plants showed no differences in dry mass at the end of the experiment, despite reductions of up to 14% in EC treatments. These results suggest that early differences internal herbicide concentrations were insufficient to cause severe damage, and exposure did not exceed the physiological tolerance of soybean at the evaluated growth stage. In crops like rice and corn, similar responses have been reported and are associated with detoxification and physiological acclimation.^37^

Finally, this study did not aim to evaluate weed control by the different clomazone formulations, as previous research has demonstrated that ME formulations maintain herbicide efficacy.^14,20,21^ Instead, this work provides a comparative assessment of formulation-related differences in herbicide dynamics across soil, residue, and plant compartments. The results highlight consistent differences between the evaluated formulations, although interpretations should remain limited to the products tested.

## 5 Conclusion

The evaluated ME clomazone formulations altered the environmental dynamics of the herbicide by promoting a gradual release of the active ingredient in soil and crop residue under the experimental conditions. Compared to EC formulations, ME formulations showed lower initial losses and greater cumulative availability of clomazone over time.

In corn residue, ME formulations showed higher retention and persistence of clomazone under increasing rainfall, which is consistent with reduced wash-off under the evaluated conditions. This behavior may favor herbicide transfer to the soil during precipitation events. In soybean plants, ME formulations limited foliar absorption and internal exposure, maintaining most of the herbicide on the leaf surface and reducing transient effects on plant growth. This delayed internal availability is consistent with the greater crop selectivity observed compared with the tested EC formulations.

The results indicate that the evaluated ME formulations may improve clomazone stability, crop exposure, and selectivity under the conditions of this study. However, these findings are limited to the specific formulations evaluated and should not be generalized to all microencapsulated or EC technologies.

## Supplementary Material



## Notes

Analytical calibration curves and coefficients of determination for clomazone and its metabolites (Table S1), molecular masses and fragment ions used for LC-MS/MS analyses (Table S2), ANOVA summary tables for clomazone leaching data (Table S3), and representative chromatograms of clomazone, hydroxyclomazone, and ketoclomazone (Figure S1) (PDF)

## References

[1] CONAB – Companhia Nacional de Abastecimento Nova estimativa da Conab para safra de grãos 2024/25 É de 322,53 milhões de toneladas; CONAB: Brasília, 2024. https://www.conab.gov.br/ultimas-noticias/5821-nova-estimativa-da-conab-para-safra-de-graos-2024-25-e-de-322-53-milhoes-de-toneladas. (accessed 23 March 2025).

[2] FEBRAPDP – Federação Brasileira de Plantio Direto na Palha Superfície sob plantio direto 2019. https://febrapdp.org.br/download/area-PD-Brasil-e-estados.pdf.

[3] Fuentes-Llanillo R., Telles T. S., Junior D. S., De Melo T. R., Friedrich T., Kassam A. (2021). Expansion of no-tillage
practice in conservation agriculture
in Brazil. Soil Tillage Res..

[4] Bartz M. L. C., Telles T. S., Junior R. C., Fuentes-Llanillo R., Ralisch R. (2025). No-tillage system: A genuine Brazilian technology that
meets current global demands. Adv. Agron..

[5] Risi F. G. E., Hüther C. M., Righi C. A., Umburanas R. C., Tezotto T., Dourado
Neto D., Reichardt K., Pereira C. R. (2024). Sustainability analysis of nitrogen
use efficiency
in soybean–corn succession crops of Midwest Brazil. Nitrogen.

[6] Colbach N., Adeux G., Cordeau S., Moreau D. (2023). Weed-induced yield
loss through resource competition cannot be sidelined. Trends Plant Sci..

[7] Maheswari, S. T. Use of herbicide and its implications under no-till farming: An overview. In Conservation Agriculture: A Sustainable Approach for Soil Health and Food Security; Jayaraman, S. ; Dalal, R. C. ; Patra, A. K. ; Suresh, S. ; Eds.; Springer Nature: Singapore, 2021, pp. 423–431. DOI: 10.1007/978-981-16-0827-8_21.

[8] Douibi M., Carpio M. J., Rodríguez-Cruz M. S., Sánchez-Martín M. J., Marín-Benito J. M. (2024). Control
of the field herbicide dissipation
by cover crop mulch in conservation agriculture. Agronomy.

[9] Fleming D. E., Spencer G. D., Krutz L. J. (2025). Pesticide
runoff from conventional
tillage, minimum tillage, and no-tillage cropping systems: Meta-analysis
of published North American data. J. Environ.
Qual..

[10] Álvarez F., Arena M., Auteri D., Leite S.B., Binaglia M., Castoldi A.F., Chiusolo A., Colagiorgi A., Colas M., Crivellente F., European
Food Safety Authority (EFSA) (2025). Peer review of the pesticide risk assessment of the active substance
clomazone. EFSA J..

[11] Lins H. A., de Freitas Souza M., Batista L. P., da Silva
Rodrigues L. L. L., da Silva F. D., Fernandes B. C. C., da Melo S. B., das Chagas P. S. F., Silva D. V. (2024). Artificial intelligence
for herbicide recommendation:
Case study for the use of clomazone in Brazilian soils. Smart Agric. Technol..

[12] Zargar M., Pakina E. N., Romanova E. V. (2014). Herbicide
doses and application times
in weed suppression on different red bean varieties. APCBEE Proc..

[13] PPDB – Pesticide Properties Database List of Pesticide Active Ingredients; University of Hertfordshire, 2025. https://sitem.herts.ac.uk/aeru/ppdb/en/atoz_herb.htm.

[14] Tropaldi L., Brito I. P. F. S., Dias R. C., Trindade M. L. B., Carbonari C. A., Velini E. D. (2019). Dynamics of clomazone formulations under different
application conditions. Planta Daninha.

[15] Tropaldi L., Carbonari C. A., De Brito I. P. F., de Matos A. K. A., de
Moraes C. P., Velini E. D. (2021). Dynamics of clomazone formulations
combined with sulfentrazone in sugarcane straw. Agriculture.

[16] Singh G., Ramadass K., Sooriyakumar P., Hettithanthri O., Vithange M., Bolan N., Tavakkoli E., Van Zwieten L., Vinu A. (2022). Nanoporous materials for pesticide
formulation and delivery in the agricultural sector. J. Controlled Release.

[17] Sopeña F., Maqueda C., Morillo E. (2009). Controlled
release formulations of
herbicides based on micro-encapsulation. Cienc.
Inv. Agr..

[18] Whorton, C. Factors influencing volatile release from encapsulation matrices ACS Publications ACS Symposium Series 1995 590 134–142 10.1021/bk-1995-0590.ch012

[19] Włodarczyk M., Siwek H. (2016). Influence of formulation on mobility
of clomazone in soil. Bull. Environ. Contam.
Toxicol..

[20] Hennens D., Sarazin M., Casaña-Giner V., Gimeno M. (2014). Advanced formulation
technology and its benefits for clomazone-containing herbicides. Julius-Kühn-Arch.

[21] Keifer D. W., Dexter R. W., Nicholson P., Pepper R. F. (2007). Microencapsulated
clomazone: Formulation stability, tank mix volatility, and solvent
effects. J. ASTM Int..

[22] Mitscherlich, E. A. Das gesetz des minimums und das gesetz des abnehmenden bodenertrages Landwirtsch Jahrbuch 1909 38 537–552

[23] Fernández-Pérez M. (2007). Controlled
Release of Pesticides from Microcapsules. J.
Environ. Sci. Health, Part B.

[24] Silva P. T., Fries L. L. M., Menezes C. R. D., Holkem A. T., Schwan C. L., Wigmann É. F., Bastos J. O., Silva C. D. B. D. (2014). Microencapsulation:
Concepts, mechanisms, methods, and applications in food technology. Cienc. Rural.

[25] Sabarivasan R., Arthanari P. M. (2025). Application of Nanoencapsulation
Technology in Agriculture
for Effective and Sustainable Weed Management: A Critical Review. Commun. Soil Sci. Plant Anal..

[26] Ferhatoglu Y., Barrett M. (2006). Studies of clomazone mode of action. Pestic. Biochem. Physiol..

[27] Gish T. J., Shirmohammadi A., McConnell L. L. (2011). Volatilization
Losses of Pesticides
from Soil and Plant Surfaces. J. Environ. Qual..

[28] Sopeña F., Villaverde J., Maqueda C., Morillo E. (2011). Photostabilization
of norflurazon microencapsulated with ethyl-cellulose. J. Hazard. Mater..

[29] Oliveira M. F. D., Alvarenga R. C., Oliveira A. C. D., Cruz J. C. (2001). Effect
of corn residue
and atrazine plus metolachlor mixture on weed control in no-till corn. Pesqui. Agropecu. Bras..

[30] Carbonari C. A., de Matos A. K. A., De
Brito I. P. F. S., Velini E. D., Dayan F. E. (2020). Impact
of green cane harvesting on pest management in sugarcane. Outlooks Pest Manag.

[31] Mervosh T. L., Sims G. K., Stoller E. W. (1995). Clomazone fate in soil as affected
by microbial activity, temperature, and moisture. J. Agric. Food Chem..

[32] Salzman F. P., Renner K. A., Penner D. (1992). Absorption, translocation, and metabolism
of clomazone, metribuzin, and linuron in soybean and cocklebur. Weed Sci..

[33] Mendes, K. F. ; Mielke, K. C. ; D’Antonino, L. ; da Silva, A. A. Retention, absorption, translocation, and metabolism of herbicides in plants. In Applied Weed and Herbicide Science; Mendes, K. F. ; da Silva, A. A. ; Eds.; Springer International Publishing: Cham, 2022, pp. 157–186 10.1007/978-3-031-01938-8_5.

[34] Alcántara-de la Cruz, R. ; da Silva Amaral, G. ; Mendes, K. F. ; Rojano-Delgado, A. M. ; De Prado, R. Absorption, translocation, and metabolism studies of herbicides in weeds and crops. In Radioisotopes in Weed Research; Mendes, K. F. ; Ed.; CRC Press: Boca Raton, FL, 2020, pp. 127–154 10.1201/9781003005070-5.

[35] Norman M. A., Liebl R. A., Widholm J. M. (1990). Uptake and metabolism
of clomazone
in tolerant soybean and susceptible cotton photomixotrophic cell suspension
cultures. Plant Physiol..

[36] Gupta, A. ; Tripathy, D. B. ; Kumar, G. ; Agarwal, P. ; Ghosal, A. Nanopesticides, Nanoherbicides, and Nanofertilizers: Formulations and Applications; CRC Press, Taylor & Francis Group: Boca Raton, FL, 2023 10.1201/9781003364429.

[37] Schreiber F., Avila L. A., Scherner A., Gehrke V. R., Agostinetto D. (2015). Volatility
of different clomazone formulations. Planta
Daninha.

