# Supplementary material for: Microencapsulated and Conventional Clomazone Formulations: Dynamics in Soil, Crop Residue, and Soybean
Source: ACS Omega. 2026 Jun 11;11(25):37113–21. doi: 10.1021/acsomega.6c01359 (PMC13325070; doi:10.1021/acsomega.6c01359)
Supplement: Supplementary file 1 [file ao6c01359_si_001.pdf]

Supplementary material: *Microencapsulated and Conventional Clomazone Formulations: Dynamics in Soil. Crop Residue. and Soybean*

Jéssica Alves Bonamichi [1,2], Roberto Estêvão Bragion de Toledo [2], Grazielle Rodrigues Araujo [3]*, Ana Karollyna Alves de Matos [4], Ivana Paula Ferraz Santos de Brito [4], Diego Gonçalves Alonso [2], Richard Feliciano [2], Ricardo Alcántara-de la Cruz [3]*, Edivaldo D. Velini [1], Caio A. Carbonari [1]

Table S1. Analytical curve of clomazone. metabolites and their respective coefficients of determination

Compound	Analytical curve	Coefficient of determination (r^2)
Clomazone	y = 460.581.1x + 1.289.94	0.9993
Hydroxyclomazone	y = 173.498.9x – 3.927.553	0.9999
Ketoclomazone	y = 5.996.122x + 3.382.507	0.9974

Table S2. Molecular mass and fragments of clomazone and its analyzed metabolites

Compound	Molecular mass	Fragment
Clomazone	240.200	125.100<br>89.100<br>99.100
Hydroxyclomazone	256.062	125.000<br>89.200<br>63.200
Ketoclomazone	254.062	125.000<br>89.200<br>63.200

Table S3. Summary of ANOVA and mean comparison test for percentage of clomazone leaching from different formulations applied to medium-textured soil as a function of rainfall timing after application

**Product**	**Rainfall occurrence period after application (days)**				
	**1**	**3**	**7**	**14**	**30**
Leached clomazone – free fraction (% of applied dose)					
ME-1	5.3 Ce*	3.9 Cd	15.2 Aa	10.8 Ab	7.2 Ac
ME-2	4.5 Cc	4.2 Abc	14.0 Aa	8.8 Bb	5.4 Bc
EC-1	21.8 Aa	5.3 Ab	3.5 Bc	2.1 Cd	1.5 Cd
EC-2	19.7 Aa	4.0 ABb	3.0 Bbc	1.9 Ccd	1.1 Cd
F Product	24.659**				
F Period	250.681**				
F Product * Period	170.37**				
CV (%)	13.2				
Encapsulated clomazone leached (% of applied dose)					
ME-1	0.071 Aa	0.115 Aa	0.113 Aa	0.066 Aa	0.085 Aa
ME-2	0.052 Ac	0.123 Aa	0.111 Aab	0.058 Abc	0.104 Aabc
F Product	0.002^{ns}				
F Period	3.675**				
F Product * Period	0.278^{ns}				
CV (%)	44.39				
Encapsulated clomazone (% of total leached)					
ME-1	1.305 Ab	2.934 Aa	0.732 Ab	0.614 Ab	1.209 Ab
ME-2	1.152 Abc	2.916 Aa	0.779 Abc	0.669 Ac	2.023 Aab
F Product	0.269^{ns}				
F Period	8.229**				
F Product * Period	0.353^{ns}				
CV (%)	63.29				

*Means followed by the same uppercase letter in the column and lowercase letter in the row do not differ significantly according to the t-test ($p \leq 0.05$). **Significant by the F-test ($p \leq 0.05$). ns: not significant.

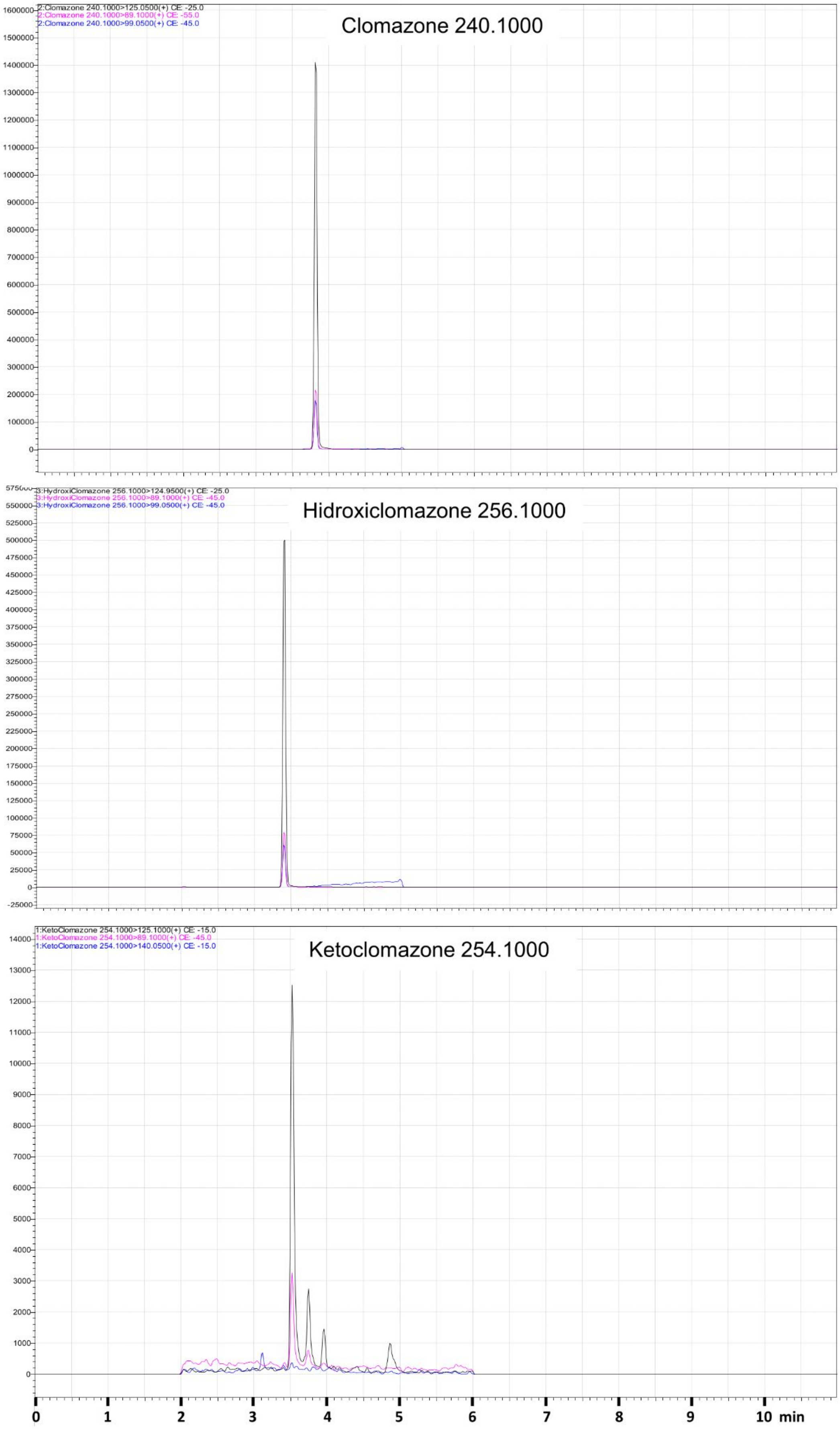

Figure S1. Chromatogram of clomazone and its metabolites hydroxyclomazone and ketoclomazone.
